# Climate Change and Health: Perspectives From Ghana

**DOI:** 10.1029/2024GH001030

**Published:** 2024-08-10

**Authors:** Martin Gameli Akakpo, Sylvia Hagan, Hayford Alufar Bokpin

**Affiliations:** ^1^ Department of Psychology Regent University College of Science and Technology Accra Ghana; ^2^ Department of Psychology University of Ghana Accra Ghana; ^3^ Centre for Migration Studies University of Ghana Accra Ghana

**Keywords:** climate change, climate injustice, sub‐Saharan Africa, health, disease

## Abstract

Climate change is impacting many aspects of human life in many ways. In Ghana, climate change knowledge remains low and discussions linking climate change and health are scarce. In this paper, authors contribute to the shaping of discussions about climate and health with a focus on how climate change increases certain ailments. First, the paper addresses the need for research in Ghanaian communities to link climate change and health. Second, the paper suggests the development of policies to address the link. Third, public health educators are advised in this paper to educate the public.

## Key Points

- Climate change is an important driver of negative health outcomes
- Climate change exacerbates the already precarious health inequality in Ghana
- Researchers, policymakers, and public health educators should focus on the impact of climate change on health in Ghana

## 1 Introduction

Globally, the link between climate change and health has long been suggested (George et al., 2024; Paavola, 2017; Patz & Olson, 2006; Romanello et al., 2023). Patz and Olson (2006) recall that the World Health Organization (WHO), has recorded more than 150,000 climate change‐related deaths since 1970. However, the focus of climate change researchers and policymakers has been primarily on treating it as an environmental issue. This leads to an incomplete understanding of the health risks posed by climate change. Increased flooding, unusual heat, rising sea levels, and droughts lead to health conditions such as diarrhea, malnutrition, communicable diseases, respiratory tract infections and some cancers (Angmor, 2024; El‐Sayed & Kamel, 2020). In Ghana, a systematic review by Nti et al. (2019), noted that most studies discuss the link between extreme weather conditions and health without clarifying that these extreme conditions are more frequent because of climate change. Hence, there is an urgent need for research and action to educate the public about the link between climate change and health in Ghana (Hussey & Arku, 2019).

Social inequality is worsened by climate change, and this impacts the health of vulnerable people (Kim et al., 2014; Peters & Kusimi, 2023). This form of climate injustice has led to the disruption of life in many vulnerable communities in sub‐Saharan African countries where health centers are already burdened (Coates et al., 2020). This paper extends calls by some researchers (George et al., 2024; Paavola, 2017; Patz & Olson, 2006) by emphasizing the urgent need for all stakeholders to include health in any discussion of and mitigation measures against climate change in Ghana. This is done by discussing the link between climate change and health in Ghana and analyzing the role played by social inequality in worsening the impact of climate change on health.

This study employed a desk review methodology, analyzing academic papers from Google Scholar, Scopus, PubMed, and Web of Science. Keywords such as “Climate change” AND “health” OR “Climate‐related diseases” AND “Ghana” were used. Relevant studies were analyzed using thematic analysis. Drawing from discussions in other countries, this paper makes suggestions to help manage the health‐linked impacts of climate change.

## 2 Climate and Health

Climate change has been directly linked to the increase in certain diseases (Paavola, 2017). Increased pollution, drought, more intense heat waves, and disruption of healthcare systems are all examples of the impact climate change can have on health (Kotcher et al., 2021). In this paper, we analyze this through two broad spectrums which are direct and indirect, an approach supported by Kim et al. (2014) and Coates et al. (2020).

The direct relationship is because adverse climate conditions such as increased frequency of floods, and droughts result in waterborne diseases, respiratory illness from increased dust and malnutrition. For instance, climate change increases the intensity and frequency of droughts which leads to an increase in dust in affected communities and this can lead to an increase in respiratory illnesses. Flooding has also been cited as a major source of water‐borne diseases (Angmor, 2024; Patz & Olson, 2006). The Lancet Countdown (Romanello et al., 2023) notes that heat‐related deaths have been at a record high since 2022 and heatwaves have been more frequent and intense in 2023 than in the past 100, 000 years.

The indirect relationship is about factors like irregular migration, and displaced settlements. Irregular migration can lead to unplanned settlements where diseases spread quickly, lack of protection for people with health vulnerabilities and forced displacement to locations with vectors (Coates et al., 2020; Kim et al., 2014). Climate change has influenced the rise in unplanned mass migration of people whose natural habitats can no longer support their lives.

In the healthcare system of most countries in sub‐Saharan Africa including Ghana, service providers do not adequately collaborate with climate scientists to discuss the change in the morbidity of certain diseases (Tosam & Mbih, 2015). This ongoing disconnect is leading to an increased burden on the already stretched healthcare system of most sub‐Saharan African countries (Coates et al., 2020).

## 3 Climate Change, Inequality and Health

Before the current wave of climate change research, political action and financial preparedness, inequality existed and may still exist even if climate change is controlled. The increase and spread of inequality are addressed in this paper.

This climate injustice leads to the poor and vulnerable bearing a heavier burden of climate change despite contributing the least toward its occurrence (Coates et al., 2020). Climate change is worsening inequality in sub‐Saharan Africa and drawing more people into poverty (Tosam & Mbih, 2015). In the context of this paper, inequality refers to the unequal access of people to income, security, housing, health, and other public services.

In sub‐Saharan African countries like Ghana, access to health care is already poor in rural communities. People are then forced to travel many more kilometers on deplorable roads partly worsened by floods, to the next health center. For instance, on the Ghanaian coast of Keta, increased severity, and incidence of coastal erosion from tidal waves have wiped away rural infrastructure and forced people into make‐shift villages with no amenities (Adu‐Gyamfi et al., 2020; Peters & Kusimi, 2023).

This overcrowding and lack of planning leads to a rise in water‐borne diseases and the rapid spread of vectors (Coates et al., 2020). People who once owned a house and used clean water have been forced to settle in camps and use stagnant water. This describes how individuals can fall into extreme poverty because of climate change. In the above case using the community of Keta in Ghana's Volta region, coastal erosion was recorded in previous decades, but the occurrence was rare and only happened once in several years. In recent years however, dangerous tidal waves erode the beach many times a year, leaving locals stranded and in need of immediate migration (Adu‐Gyamfi et al., 2020). These people also lose their livelihoods such as vegetable farms and fishing boats.

In the above‐described instance, families within a country become more unequal and a section of the population loses access to important amenities. Wealthier individuals in metropolitan areas and developed countries can afford planned migration and increased medical bills. However, the vulnerable who contributed little to the crises, bear a heavier burden (Afokpe et al., 2022; Kim et al., 2014).

The inability of public health officials to reach remote communities in time disrupts surveillance and delays the deployment of resources to prevent or control diseases.

This calls for urgent action to investigate the link between climate change and health in rural Ghanaian communities and propose practical policies to help locals mitigate climate change. This will in turn help them avoid or manage resulting adverse health outcomes.

## 4 Implications

Discussions in this paper and the call to action have implications for researchers, policymakers, and public health educators.

For researchers, the paper recommends the conduct of local studies to guide mitigation measures. Climate change studies from countries with more advanced health systems and well‐funded national resilience measures are insufficient to help poor communities in Ghana. Exposure in sub‐Saharan Africa, particularly Ghana, is extreme but national and local mitigation measures are either poorly funded or non‐existent. Researchers are called to use robust methods to generate data linking climate change and health in local communities (Angmor, 2024). Papers should then propose feasible policy measures that can be used to improve the livelihoods of people who are already vulnerable. For example, researchers can visit several flood sites over a period to record cases of diseases and compare pre‐flood and post‐flood data. This can then be linked to long‐term data to find out whether an increase in floods is associated with a rise in certain diseases. These findings should be disseminated through multiple channels including classrooms, the media, social media, and academic conferences.

For policymakers, the study recommends a shift from treating climate change as exclusively an environmental issue that can be addressed through pro‐environmental behavior, emission taxes, large climate conferences, and political promises. The paper calls for action in the form of evidence‐driven resilience measures in vulnerable communities such as those living near coasts, drought‐prone areas, and riverside settlers. For example, policymakers can investigate the mitigation measures used by coastal dwellers in Ghana and support them to own these “solutions” which work to reduce post‐disaster health risks (Afokpe et al., 2022). These measures can include sea defense systems, nature‐based solutions, planning of temporary camps, improving access to remote communities, and educating the public on how to manage climate‐linked health risks.

Public health educators play an important role in the dissemination of health information and the shaping of behavior in communities (Udoudom,et al., 2024). Discussions in the paper advise educators to sensitize the public about the health risks posed by climate change. Educators can draw from the relatively successful campaigns promoting antenatal care and HIV prevention in Ghana. In sensitizing communities, clarity about the ongoing effects of climate change should be central. Further to that, communities should be informed of the need to prepare for events for which they are at risk.

## 5 Conclusion

The paper has provided reasons for the need to the consideration of the impact climate change has on the health of residents in Ghana. The health systems which are already under immense stress are further burdened by climate change. Studies and policies on climate change continue to predominantly treat it as an environmental issue. However, some research continues to point out the link between climate change and health. Inaction can lead to worse consequences such as increased disease burden and an overwhelmed health system. There is a need for urgent action through locally conducted research by academics, comprehensive policies by local officials and feasible information dissemination by public health educators.

## Conflict of Interest

The authors declare no conflicts of interest relevant to this study.

## Data Availability

Data were not used, nor created for this research.
